# Running on the Frontline of Cardiovascular Medicine, Science, and Technology

**DOI:** 10.3389/fcvm.2020.613344

**Published:** 2021-01-20

**Authors:** Yong-Jian Geng

**Affiliations:** Division of Cardiovascular Medicine, Department of Internal Medicine, The Center for Cardiovascular Biology and Atherosclerosis Research, McGovern School of Medicine, University of Texas Health Science Center at Houston, Houston, TX, United States

**Keywords:** James T. Willerson, cardiologist, leader, collaborator, friend

## Introduction

On Wednesday, September 16, 2020, I received the shocking and sad news that James T. Willerson, MD, had passed away. Like many physicians, scientists, and administrators who have been mentored by and worked with Dr. Willerson, I found it difficult to bear the reality that we have lost an outstanding leader, cardiologist, researcher, mentor, collaborator, and friend. Here, in this opinion article, I wish to go through Dr. Willerson's career and share my own experience of how Dr. Willerson and I came to closely work together and how we became more than just collaborators for more than 20 years.

## Acquaintance of Dr. Willerson

I first heard Dr. Willerson's name in the 1970s when I was studying as a medical student in Suzhou Medical College, China. I participated in a pathophysiological journal club in which medical students were required to read medical literature recommended by their teachers. A couple of Dr. Willerson's publications in the 1970s−1980s were on the list of pioneering publications that highly impacted the medical field and were recommended by our teachers. I recalled that I was very interested in one of his publications concerning the regulatory impact of calcium ion on the inotropic actions of certain factors, such as hyperosmotic agents, norepinephrine, and paired electrical stimulation, on the heart under various pathophysiological conditions (1). I was very curious and amazed by the report from his research team, which illustrated computed tomography for assessing acute myocardial infarcts (2). At that time, I never thought I'd work closely with Dr. Willerson 1 day.

I met Dr. Willerson in person for the first time many years later when I participated in an annual meeting of the American Heart Association (AHA) in the 1990s. Then, I was doing research on inflammation and apoptosis in atherosclerosis with my mentor Dr. Peter Libby at the Unit of Vascular Medicine, Brigham and Women's Hospital, Harvard Medical School, Boston. Dr. Willerson came to my presentation and asked several questions, softly and in a very friendly manner, about the role of inflammatory factors in the regulation of vascular cell apoptosis and proliferation. In 1998, I received a phone call from Houston and immediately recognized Dr. Willerson's characteristic soft voice that was so deeply engrained in my memory since I met him several years ago. The phone call turned out to be a remote interview for a faculty position in cardiology at the McGovern School of Medicine, University of Texas Health Science Center at Houston (UTHealth). He and I had an inspiring conversation on various aspects of science and life from a different cultural background. In the end, luckily and honorably, Dr. Willerson kindly proposed a job offer, and I gladly accepted it. Before the end of 1998, I came to Houston and met Dr. Willerson again in person. I was very much inspired by his vision and determination to develop a world-class research program for exploring cellular and molecular cardiovascular inflammation, atherosclerosis, heart failure, and regenerative medicine. Those were topics that I was interested in and had put in tremendous effort and time to study. Since the 1998 meeting with Dr. Willerson, my tenure with UTHealth and the Texas Heart Institute (THI) and collaboration with Dr. Willerson of more than 20 years had begun.

## Early Career of Dr. Willerson

Dr. Willerson was born to a medical family in Lampasas, Texas, and grew up in San Antonio, Texas, where he attended the San Antonio Academy grade school and The Texas Military Academy (TMI) high school. During his senior year at TMI, he served as a battalion commander, president of his class, and editor of the school newspaper. He obtained a state swimming championship by winning five first places in the state private school swim meet. He entered and received a BA at the University of Texas Austin, Texas, in 1961, and moved to medical study at the Baylor College of Medicine, Houston, Texas. He received an MD in 1965. In 1967–1969, he trained as a resident of internal medicine at Massachusetts General Hospital, Boston, and then clinical associate at the National Institutes of Health, Bethesda, Maryland (1969–1972). Thereafter, he returned to Boston and finished his research and clinical fellowship at the Cardiac Unit, Department of Medicine, Massachusetts General Hospital (1972–1973).

## Professional Leadership and Achievements

He was recruited back to Texas and became an assistant professor of medicine, University of Texas Southwestern Medical School, Dallas, Texas (1973–1976). He was promptly promoted to associate and then full professor of medicine and served as Chief of Cardiology, Parkland Memorial Hospital, Dallas, Texas (1975–1989). In 1989, Dr. Willerson became a distinguished faculty member at UTHealth, entitled Edward Randall III Professor, chairman of the Department of Internal Medicine at the University of Texas-Houston Medical School, and chief of the Medical Service at Hermann Hospital (1989–2001). In March 2001, Dr. Willerson was appointed as the president of UTHealth, and he held the title of Alkek-Williams Distinguished Professor. Under his leadership, UTHealth expanded rapidly, with six buildings for education, research, and clinical care added to its campus.

Dr. Willerson held many important positions as an administrator. In recent years, he served as the president and president emeritus, director of Cardiology Research, and codirector of the Cullen Cardiovascular Research Laboratories at THI at CHI St. Luke's Health-Baylor St. Luke's. From 2001 to 2008, he served as president of UTHealth, and he recently retired as the Edward Randall III Professor of Internal Medicine at the UT Medical School at Houston. He held the Dunn Chair in Cardiology Research at THI, the Willerson/O'Quinn Chair at THI, the James T. Willerson, MD, Distinguished Chair in Cardiovascular Diseases at UT Southwestern Medical School in Dallas, and The Institute of Molecular Medicine at UTHealth. He was named a distinguished alumnus at the University of Texas, Austin, and at Baylor College of Medicine. A swimming scholarship is named in his honor at The University of Texas at Austin.

In 1993, Dr. Willerson served as the editor-in-chief of *Circulation*, a leading professional journal of the AHA. He held the editor position for 11 years, the longest tenure in the journal's editorial history. He edited or coedited 27 textbooks and published more than 1,000 scientific articles in major scientific journals. He was visiting professor and invited lecturer at more than 260 institutions worldwide.

Dr. Willerson's academic achievements have been well-recognized. He received many awards and honors from domestic and international institutions and organizations. To name some of them, the following is a brief list of the awards and honors that Dr. Willerson received in the past two decades.

1993, the James B. Herrick Award” from AHA; 2000, Distinguished Scientist Award from the American College of Cardiology; 2002, the Distinguished Achievement Award, AHA; 2003, Distinguished Scientist Award, AHA; 2005, the Gold Heart Award, AHA; Fellow of the Royal Society of Medicine, the United Kingdom; Honorary Member of 10 foreign Societies of Cardiology; Member and past President of the Paul Dudley White Cardiology Society at Massachusetts General Hospital; 2004, the Medal of Merit for Distinguished Achievements in Cardiovascular Sciences by the International Academy of Cardiovascular Sciences; 2005, the “Lifetime Achievement Award”, the Cardiovascular Research Foundation. 2006, the Libin Award in Cardiovascular research, Canada; 2006, the “Living Legend Award” for achievement in cardiovascular research, the World Society of Cardiothoracic Surgeons; 2007, the Katz Research Prize from Columbia University College of Physicians and Surgeons, New York; 2011–2014, President of the International Society for Cardiovascular Sciences, Canada; 2009–2010, President of the Board of the American Heart Association, Houston Chapter; 2012, the Lifetime Achievement Award, the All India Institute of Medical Sciences; 2014, Chair of the Scientific Advisory Board of the Trans-Atlantic Network of Excellence of the Leducq Foundation.

## Physician, Educator, and Scientist

Dr. Willerson was an internationally recognized cardiologist, who always put his patients first. In the cardiology clinic of St. Luke's Hospital-THI, he had a medical practice and service for more than 3,000 patients. He saw patients daily until shortly before becoming very ill and hospitalized. Dr. Willerson was wholeheartedly devoted to his patients and provided them with exemplary care. During the COVID-19 pandemic, he was working at the hospital and saw patients until shortly before his final illness. Dr. Willerson always put his patients first. For the past 20 years, many of my meetings with Dr. Willerson took place in his clinic rather than in an administrative office so that he could have more time for taking care of his patients. I traveled together with Dr. Willerson to attend many domestic and international conferences. I noticed that he was often on the phone chatting with his patients and/or his clinical staff or partners to discuss and decide the diagnosis and therapeutic strategy or to offer medical consultation. It was not uncommon that he responded simultaneously to different patient's phone calls.

Dr. Willerson was an exceptional medical educator. He demonstrated to medical students, residents, fellows, and junior faculty members his mastery of clinical history and physical examination in ward rounds and morning case reports, a memorable event where proper preparation by residents is mandatory. Dr. Willerson's lectures were always evidence-based and presented logically and in a good charity. He served on numerous editorial boards for professional publications, publishing his own signature textbook, the third edition of *Cardiovascular Medicine*, released in February 2007.

Dr. Willerson was also an outstanding investigator. His research was concentrated on the detection and treatment of unstable atherosclerotic plaques and the discovery of the genes and abnormal proteins responsible for cardiovascular disease. For the past two decades, he led a pioneering research project that was directly involved in seminal research into the use of stem cells for the repair of hearts and cardiovascular vessels injured by heart attacks. These landmark discoveries led to THI being awarded the first US Food and Drug Administration (FDA)–approved human clinical trial using adult, human stem cells to treat ischemic cardiomyopathies and congestive heart failure. His accolades and awards represent the highest achievement in cardiology practice, research, and leadership at a global level.

## Mentor, Collaborator, and Friend

**Figure 1:**
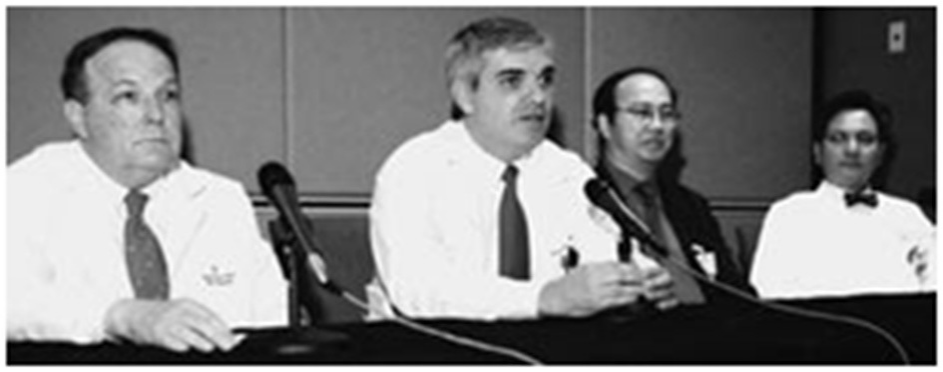
Dr. James T. Willerson leading a stem cell clinical trial for heart failure. UTHealth and THI clinical trial leaders in a press conference, left to right: James T. Willerson, MD; Emerson Perin, MD, PhD; Yong-Jian Geng, MD, PhD; and Edward T. H. Yeh, MD, at the news conference announcing FDA approval of a stem cell clinical trial for heart failure (The Leader, April 2004).

Dr. Willerson was a truly visionary leader running on the frontline of medicine, science, and technology. He was always enthusiastic about new developments of medicine, science, and technology. He encouraged his colleagues to seek novel research ideas, especially in stem cell biology and regenerative medicine. I recalled how excited he was when he saw beating heart muscle cells derived from stem cells under an inverted microscope in the tissue culture room. Dr. Willerson, Dr. Emerson Perin (now medical director of THI), and I (Figure 1) were working together to quickly assemble a research team with experts who were capable of performing both basic and clinical stem cell research. We conducted, for the first time, a clinical trial for treatment of end-stage heart failure with adult bone marrow cells, through transendocardial, autologous bone marrow cell transplantation with a sophisticated catheter-based delivery system (3). Dr. Willerson and I developed small- and large-animal models of vascular injury and myocardial infarction, and we explored the potential of stem cell therapy in regenerative cardiovascular medicine (4). Under his guidance and leadership, in the 2000s, our research team performed multiple experiments, examining cardiovascular stem cell functions in various animal models of myocardial infarction and ischemic heart failure. We demonstrated that mesenchymal stem cells can boost endothelial function in a canine infarct model (5) and the existence of cardiovascular myogenic stem cells in murine adipose tissue (6). We showed that interplays between myocardin and telomerase mediate the cardiovascular myogenic development of stromal cells from adipose tissue (7). We further illustrated that in murine limb ischemia, stem cells with a co-expression of two nuclear factors are rejuvenated possessing a high potency of repair and generating cardiovascular tissues damaged by ischemia (8). Collaborating with Dr. Willerson, our research team identified several factors potentially regulating stem cell growth and differentiation. Among them are the cholesterol-lowering drug statins (9) and the hormone erythropoietin (10).

Dr. Willerson always treated his students or trainees respectfully. Throughout his career, Dr. Willerson mentored numerous junior faculty members, residents, and fellows as well as research scientists regardless of their race and ethnic origins. For instance, Song Gao (PhD, from China) and Harnath Shelat (MS, from India) are two investigators of our research group who have worked for the past decade on several projects collaborating with and advised by Dr. Willerson. Very often, Dr. Willerson greeted young investigators warmly and offered advice or suggestions whenever there was chance of meeting. Dr. Willerson took care of people who worked in the clinic as well as research laboratories. Indeed, Dr. Willerson had the warmest heart and respected his colleagues. He treated his students and coworkers like his family members. Each time when we ran into each other, Dr. Willerson always asked after my family's well-being and how they were doing. In the first 10 years of my life in Houston, I received his greeting card and flowers delivered to my home address every year for the Christmas/New Year holidays.

We will forever remember Dr. Willerson for his leadership, mentorship, and friendship.

## Author Contributions

The author confirms being the sole contributor of this work and has approved it for publication.

## Conflict of Interest

The author declares that the research was conducted in the absence of any commercial or financial relationships that could be construed as a potential conflict of interest.
